# HER2-Targeted Tyrosine Kinase Inhibitors Cause Therapy-Induced-Senescence in Breast Cancer Cells

**DOI:** 10.3390/cancers11020197

**Published:** 2019-02-08

**Authors:** Martina S. J. McDermott, Neil Conlon, Brigid C. Browne, Adam Szabo, Naoise C. Synnott, Neil A. O’Brien, Michael J. Duffy, John Crown, Norma O’Donovan

**Affiliations:** 1 Division of Hematology-Oncology, Department of Medicine, David Geffen School of Medicine, University of California at Los Angeles, Los Angeles, CA 90095, USA; NOBrien@mednet.ucla.edu; 2 Molecular Therapeutics for Cancer Ireland, National Institute for Cellular Biotechnology, Dublin City University, Glasnevin, 9 Dublin, Ireland; neil.conlon@dcu.ie (N.C.); brigid_browne@yahoo.com (B.C.B.); adamkoszabo@gmail.com (A.S.); john.crown@cancertrials.ie (J.C.); norma.odonovan@dcu.ie (N.O.); 3 UCD School of Medicine and Medical Science, UCD Conway Institute of Biomolecular and Biomedical Research, University College Dublin, 4 Dublin, Ireland; naoisesynnott@gmail.com (N.C.S.); michael.j.duffy@ucd.ie (M.J.D.); 4 UCD Clinical Research Centre, St. Vincent’s University Hospital, 4 Dublin, Ireland; 5 Department of Medical Oncology, St. Vincent’s University Hospital, Elm Park, 4 Dublin, Ireland

**Keywords:** lapatinib, HER2, breast cancer, TKI, erbB2, senescence, neratinib, afatinib, trastuzumab

## Abstract

Prolonged treatment of HER2 positive breast cancer cells with tyrosine kinase inhibitors (TKIs) leads to the emergence of acquired resistance. However, the effects of continuous TKI exposure on cell fate, and the steps leading to the acquisition of a resistant phenotype are poorly understood. To explore this, we exposed five HER2 positive cells lines to HER2 targeted therapies for periods of up to 4 weeks and examined senescence associated β-galactosidase (SA-β-gal) activity together with additional markers of senescence. We found that lapatinib treatment resulted in phenotypic alterations consistent with a senescent phenotype and strong SA-β-gal activity in HER2-positive cell lines. Lapatinib-induced senescence was associated with elevated levels of p15 and p27 but was not dependent on the expression of p16 or p21. Restoring wild type p53 activity either by transfection or by treatment with APR-246, a molecule which reactivates mutant p53, blocked lapatinib-induced senescence and caused increased cell death. In contrast to lapatinib, SA-β-gal activity was not induced by exposing the cells to trastuzumab as a single agent but co-administration of lapatinib and trastuzumab induced senescence, as did treatment of the cells with the irreversible HER2 TKIs neratinib and afatinib. Neratinib- and afatinib-induced senescence was not reversed by removing the drug whereas lapatinib-induced senescence was reversible. In summary, therapy-induced senescence represents a novel mechanism of action of HER2 targeting agents and may be a potential pathway for the emergence of resistance.

## 1. Introduction

Human epidermal growth factor receptor 2 (HER2) is overexpressed in 20–25% of breast cancers and is associated with an aggressive phenotype and a poor clinical outcome for patients [1]. HER2 targeted therapies including monoclonal antibodies and tyrosine kinase inhibitors (TKIs) have significantly improved patient outcome, particularly for patients with early stage breast cancer. Examples include trastuzumab, a humanized monoclonal antibody that targets and binds to the extracellular domain of HER2, preventing the activation of its kinase domain [2], and lapatinib, an orally active, selective and reversible low molecular weight TKI that targets both EGFR and HER2. Lapatinib, in combination with capecitabine, is approved for the treatment of HER2-positive metastatic breast cancer that has failed prior treatment with trastuzumab [3]. Lapatinib is also approved for use in combination with letrozole in HER2- and ER-positive metastatic breast cancer [4]. Studies of short-term (1–7 days) treatment have shown that lapatinib induces cell cycle arrest, apoptosis and autophagy [5,6,7]. A recent study also reported that in addition to apoptosis, lapatinib combined with radiation resulted in the induction of senescence [8].

Senescence was first observed in vitro in primary cells, whereby replicative exhaustion resulted in irreversible senescence with telomere shortening [9], and was termed replicative senescence. Oncogene signaling can also result in senescence (oncogene induced senescence (OIS)), which acts as a tumor suppressive mechanism and a barrier to cancer progression [10]. A third type of senescence has recently been described in response to therapy, whereby cancer cells can undergo apoptosis or enter therapy-induced-senescence (TIS). The senescent cells may be cleared by the immune system, but some cancer cells can remain ‘dormant’ and potentially contribute to the development of resistance [11]. In contrast to replicative senescence and OIS, TIS occurs following therapeutic intervention and may be viewed as a therapeutic goal of drug treatment whereby persistent growth inhibition is achieved.

Several studies have shown, using a variety of cell line models, that long-term continuous lapatinib treatment (3–6 months) results in the development of acquired lapatinib resistance [12,13,14,15]. However, the effects of continuous lapatinib treatment on cell fate are not well defined. The purpose of this study was to examine the effects of continuous lapatinib exposure on the growth of HER2-positive breast cancer cells in order to examine possible mechanisms of lapatinib action and to elucidate potential mechanisms for the development of acquired resistance.

## 2. Results

### 2.1. Lapatinib Induces Senescence in HER2 Positive Breast Cancer Cell Lines

**Figure 1:**
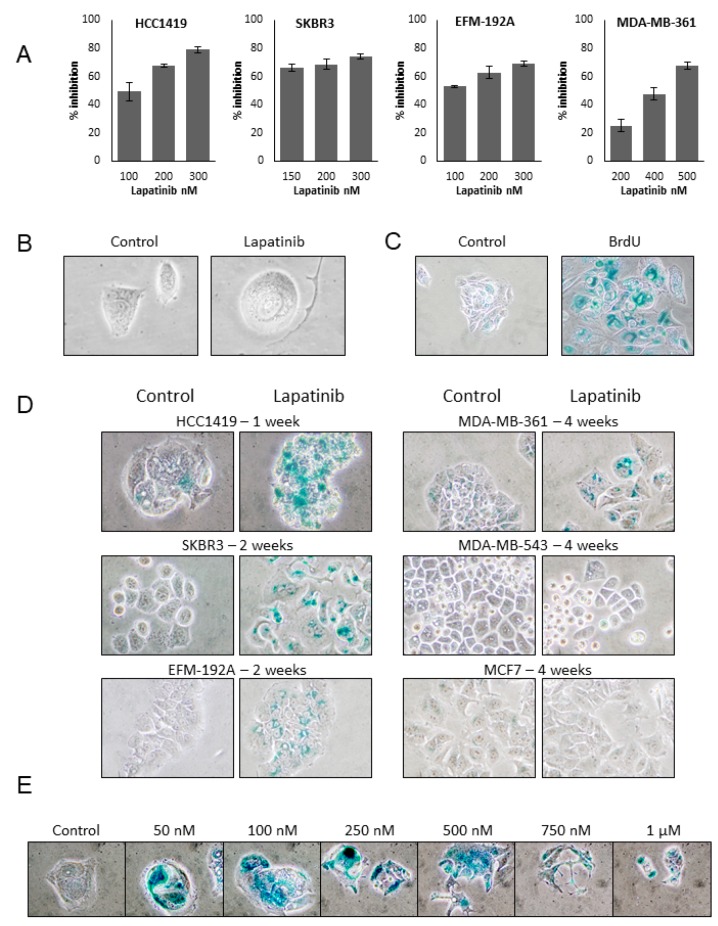
(**A**) Effect of varying concentrations of lapatinib on the growth of HER2 positive breast cancer cells. Cells were treated for 4 days and then counted using Viacount on the Guava system. The effect of lapatinib on the growth of cells was determined relative to untreated control cells (error bars reflect *n* = 3). (**B**) HCC1419 cells were treated twice weekly with 250 nM lapatinib for approx. 3 months. Images taken at 400× magnification. (**C**) HCC1419 cells were treated twice weekly with 50 µM bromodeoxyuridine (BrDU) for two weeks and fixed and stained for SA-β-gal activity and compared to untreated control cells (Images taken at 200×). (**D**) HCC1419, SKBR3 and EFM-192A cells were treated with 250 nM lapatinib, MDA-MB-361 cells were treated with 500 nM lapatinib and MDA-MB-453 cells and MCF7 cells as a negative control, were treated with 1 µM lapatinib, twice weekly for an extended period of time (ranging from 1–4 weeks). Cells were then fixed and stained for SA-β-gal activity and compared to untreated control cells. Images taken at 400× magnification. (**E**) HCC1419 cells were treated with a range of lapatinib concentrations twice a week for 1 week. Cells were then fixed and stained for SA-β-gal activity. Images taken at 400× magnification.

Four lapatinib sensitive HER2 positive cell lines (HCC1419, SKBR3, EFM-192A and MDA-MB-361), 1 lapatinib resistant HER2 positive cell line (MDA-MB-453) and a non-HER2 overexpressing cell line (MCF7) were tested for the effects of continuous lapatinib treatment. The effects of multiple doses of lapatinib treatment were measured after 4 days and an approx. lapatinib IC_70_ concentration was determined for each cell line; 250 nM for SKBR3, HCC1419 and EFM-192A, 500 nM for MDA-MB-361, while 1 µM was selected for use for MDA-MB-453 and MCF7 (Figure 1A). This concentration was selected as our previous work with lapatinib has shown that extended long term treatment of cell lines with these IC_70_ concentrations results in the emergence of lapatinib resistance [14]. The cells were then treated twice weekly with the appropriate concentration of lapatinib. Lapatinib sensitive cells exhibited distinct alterations in cellular morphology while undergoing continuous lapatinib treatment, including increased cell size and development of vacuoles which are characteristics of senescent cells [16]. While these morphologic alterations were apparent as early as 1 week after treatment they are most easily appreciated in cells undergoing long-term continuous treatment with lapatinib such as the HCC1419 cells depicted in Figure 1B, following 3 months of treatment. The most widely accepted marker of senescence is senescence associated β-galactosidase (SA-β-gal) activity [17], and two weeks of treatment with 50 µM bromodeoxyuridine (BrdU) has been shown to induce senescence in mammalian cells regardless of cell type or species and was therefore used a positive control for the induction of (SA-β-gal) activity in HCC1419 cells (Figure 1C). The lapatinib treated cells were stained weekly for SA-β-gal activity and increased SA-β-gal activity was observed in SKBR3 and EFM-192A cells after 2 weeks and in MDA-MB-361 cells after 4 weeks of lapatinib treatment (Figure 1D). HCC1419 cells exhibited strong SA-β-gal activity after only one week of treatment. Thus, HCC1419 cells were selected for further examination of the lapatinib-induced senescent phenotype. Neither MDA-MB-453 nor MCF7 cells exhibited SA-β-gal activity in response to lapatinib at any time point tested (Figure 1D). The dose-dependence of the lapatinib-induced senescent phenotype was examined by treating HCC1419 cells with a range of lapatinib concentrations from 50 nM to 1 µM for one week (Figure 1E). All concentrations of lapatinib tested resulted in SA-β-gal activity and the comparative levels of SA-β-gal activity were estimated by comparing both gray mean (Supplementary Figure S1A) and blue channel mean intensity levels (Supplementary Figure S1B) between triplicate images of control and treated cells. Interestingly, lower concentrations of lapatinib (50–500 nM) resulted in stronger SA-β-gal activity compared to higher concentrations (750 nM and 1 µM).

### 2.2. Lapatinib-Induced Senescence Is Associated with Increased p15 and p27 Expression

**Figure 2:**
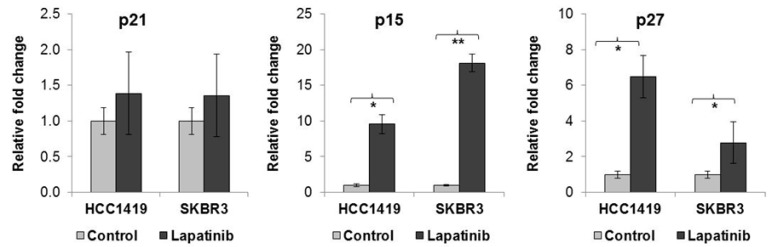
HCC1419 and SKBR3 cells were treated with 250 nM lapatinib for 1 and 2 weeks respectively, after which time RNA was isolated from control and treated cells. qRT-PCR was performed for senescence associated genes p15, p21 and p27 and the results are expressed as a fold-change in expression in lapatinib treated cells relative to untreated control cells for each cell line. * *p* < 0.005; ** *p* < 0.005 (error bars reflect *n* = 3, *p*-values calculated using Students *t*-test in excel).

The expression of senescence-associated p15^INK4b^ (p15), p16^INK4a^ (p16), p21^cip1/waf1^ (p21) and p27^Kip1^ (p27) genes increases during the induction and maintenance of senescence (reviewed in [16]). Following lapatinib treatment in both HCC1419 and SKBR3 cells, there was no significant change in p21 expression, however, p15 expression increased 9.6 ± 1.3 fold (*p* = 0.007) in HCC1419 cells and 18.1 ± 1.3 fold (*p* = 0.001) in SKBR3 cells compared to untreated cells (Figure 2). In addition, p27 mRNA levels increased 6.5 ± 1.2 fold (*p* = 0.013) in HCC1419 cells and 2.8 ± 0.4 fold (*p* = 0.01) in SKBR3 cells. Expression of p16 mRNA was not detected in HCC1419, SKBR3 or in any of the 6 cell lines used in this study (Supplementary Table S1). The increased expression of these genes, together with increased SA-β-gal activity in response to lapatinib treatment, suggests induction of senescence as a novel mechanism of lapatinib action.

### 2.3. p53 in Lapatinib-Induced Senescence

**Figure 3:**
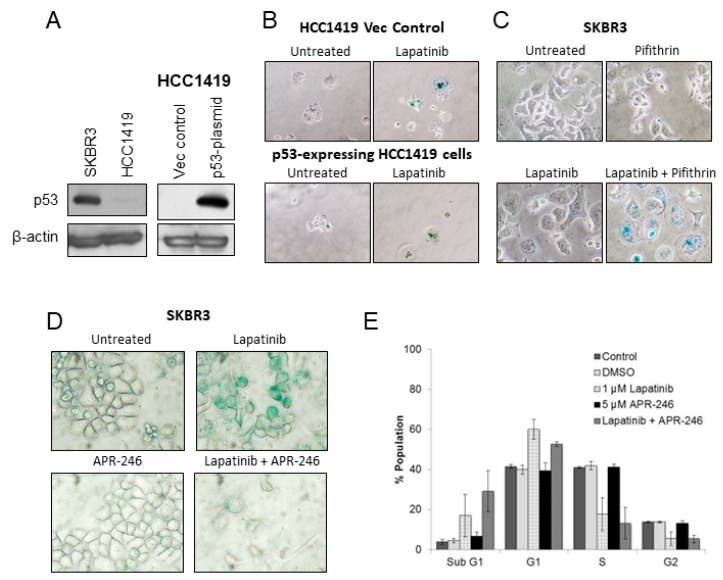
(**A**) Protein expression levels of p53 in SKBR3 and HCC1419 cells. HCC1419 cells were transfected with either empty control vector (vec control) or wild-type p53 plasmid and the resulting expression of p53 was analyzed by western blotting. β-actin was used as a loading control. (**B**) SA-β-gal activity in HCC1419 empty vector control cells and p53-expressing cells with and without 250 nM lapatinib. (**C**) SA-β-gal activity in SKBR3 cells treated with pifthrin (100 nM) alone and in combination with lapatinib (250 nM) for 1 week. (**D**) SA-β-gal activity in SKBR3 cells treated with lapatinib (250 nM) and/or APR-246 (5 µM) for 2 weeks. (**E**) Cell cycle analysis of SKBR3 cells treated with lapatinib (250 nM) and/or APR-246 (5 µM) for 5 days (error bars reflect *n* = 3). Images taken at 400× magnification.

p53 has previously been reported to be critical for the establishment of oncogene-induced senescence [18], while other studies have shown that therapy-induced senescence can occur independently of p53 [19]. Both HCC1419 and SKBR3 cell lines carry p53 mutations; HCC1419 cells are p53-null while SKBR3 cells carry a gain of function mutation (R175H) [20], and consistent with this detectable levels of p53 protein were found in SKBR3 but not HCC1419 cells (Figure 3A). Wild-type p53 was introduced into the p53-null HCC1419 cells using a pcDNA3.1 vector system resulting in detectable levels of p53 by immunoblotting (Figure 3A). In contrast to the strong SA-β-gal activity induced by lapatinib in HCC1419 p53-null cells, when wt p53-expressing HCC1419 cells were treated with lapatinib, too few viable cells remained for accurate SA-β-gal determination (Figure 3B).

SKBR3 cells were treated with lapatinib alone or in combination with the p53 inhibitor pifithrin [21] and after 1 week of treatment strong SA-β-gal activity was detected in the combination treated cells compared to either single agent, suggesting that blocking p53 activity resulted in greater induction of lapatinib-induced senescence in these cells (Figure 3C). To further examine the role of p53, SKBR3 cells were treated with lapatinib in combination with APR-246 (PRIMA-1^MET^) which is thought to act by binding to mutant p53 and restoring wt function [22]. Two weeks of lapatinib treatment induced senescence in SKBR3 cells, whereas lapatinib combined with APR-246 reduced the number of surviving cells and reduced SA-β-gal staining (Figure 3D). In cell cycle assays, lapatinib treatment resulted in increased G1 (62.5 ± 3.4%) and sub-G1 (13.5 ± 7.4%) fractions, and lapatinib in combination with APR-246 caused a lower level of G1 arrest (55.7 ± 4.3%) and an increase in the sub-G1 fraction (24.9 ± 11.85%), suggesting induction of apoptosis, however these differences did not reach statistical significance (Figure 3E).

### 2.4. Senescence is Induced by Anti-HER2 TKIs but not by Trastuzumab

**Figure 4:**
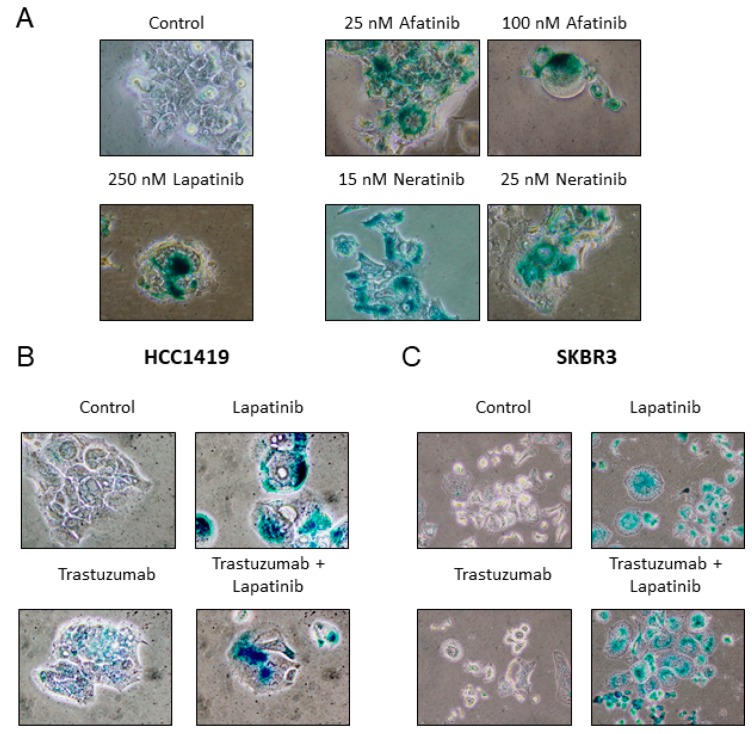
(**A**) HCC1419 cells were treated with varying concentrations of either neratinib or afatinib for 1 week. Cells were then fixed and stained for SA-β-gal activity and compared to untreated control and lapatinib treated cells. (**B**) HCC1419 cells were treated with 10 µg/mL trastuzumab alone or in combination with 250 nM lapatinib for 1 week, prior to SA-β-gal activity testing. (**C**) SKBR3 cells were treated with 10 µg/mL trastuzumab alone or in combination with 250 nM lapatinib for 2 weeks, prior to SA-β-gal activity testing. Images taken at 400× magnification.

The effects of additional HER2 targeted therapies on the induction of senescence was tested in HCC1419 cells. Interestingly, the TKIs neratinib and afatinib also induced SA-β-gal activity (Figure 4A), while treatment with the monoclonal antibody trastuzumab did not result in SA-β-gal activity (Figure 4B). However, SA-β-gal activity was induced when trastuzumab was combined with lapatinib (Figure 4B).

HCC1419 cells are innately resistant to trastuzumab in vitro, thus the effects of trastuzumab on senescence induction was also examined in the trastuzumab-sensitive SKBR3 cells. Consistent with HCC1419 cells, trastuzumab monotherapy did not induce SA-β-gal activity, however, trastuzumab with lapatinib induced SA-β-gal activity similar to lapatinib alone (Figure 4C, Supplementary Figure S2). To determine if inhibition of the MAPK and/or the PI3K/AKT pathway contributes to TKI-induced senescence, HCC1419 cells were treated with an ERK inhibitor, U0126 (MAPK inhibitor) and an AKT inhibitor, LY294002 (PI3K/AKT inhibitor). Both LY294002 (500 nM, 1 μM) and U0126 (1 μM or 2 μM) alone and in combination induced strong SA-β-gal activity (Supplementary Figure S2).

### 2.5. Lapatinib-Induced Senescence Is Reversible

**Figure 5:**
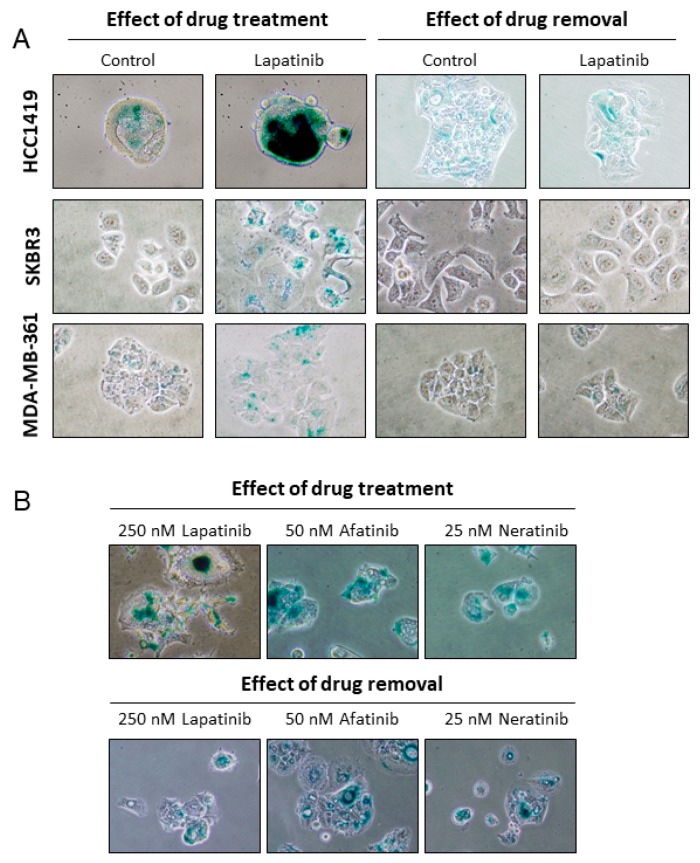
(**A**) HCC1419 cells were treated with 250 nM for 1 week, EFM-192A and SKBR3 cells were treated with 250 nM for 2 weeks and MDA-MB-361 cells were treated with 500 nM lapatinib for 4 weeks. Senescent cells were then cultured in the absence of lapatinib for 1 week prior to SA-β-gal activity testing. (**B**) HCC1419 cells were treated with the indicated concentrations of lapatinib, afatinib or neratinib for 1 week; the cells were then cultured in the absence of drug for two weeks prior to SA-β-gal activity testing. Images taken at 400× magnification.

Treatment of HCC1419 cells with lapatinib for one week induced SA-β-gal activity (Figure 1D) and continuing lapatinib treatment for a further five weeks revealed that lapatinib-induced senescence is stable while treatment is maintained; SA-β-gal activity was strongly induced at all tested time points during continuous treatment (Supplementary Figure S3). However, removal of lapatinib for 1 week resulted in decreased SA-β-gal activity, similar to levels observed in lapatinib-naïve cells. This reversal of the senescent phenotype was observed in all cell lines tested (Figure 5A). In contrast, the senescence phenotype induced in HCC1419 cells following afatinib or neratinib treatment was not reversed when the inhibitors were removed even after two weeks (Figure 5B).

**Figure 6:**
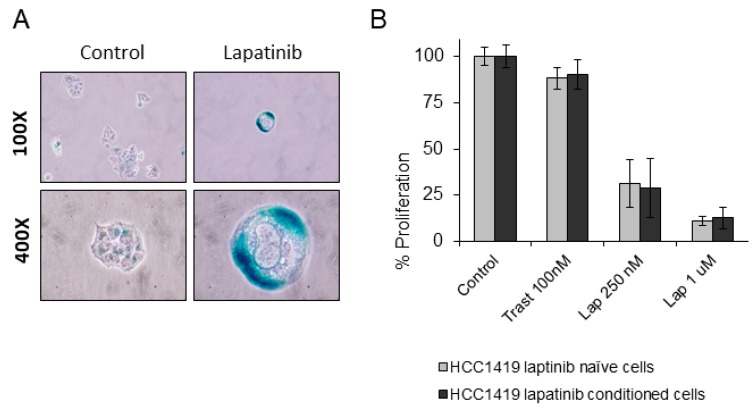
(**A**) HCC1419 cells were treated twice weekly with 250 nM lapatinib for 6 months. The resulting cells were tested for SA-β-gal activity. Images taken at 100× and 400× magnification. (**B**) The effects of trastuzumab and high and low dose lapatinib on HCC1419 cells which were lapatinib naïve or which had been previously conditioned for 6 months with 250 nM lapatinib followed by 3 months growth in lapatinib-free media. Cells were treated with the indicated concentrations of drug and then growth was measured after 5 days and is expressed relative to untreated control cells (error bars reflect *n* = 3).

To test the effects of long-term continuous lapatinib treatment, HCC1419 cells were treated with 250 nM lapatinib (twice weekly) for 6 months. All remaining cells after 6 months were senescent, exhibiting strong SA-β-gal activity and extremely large vacuoles (Figure 6A). The senescent cells were then cultured in the absence of lapatinib and the cells began to actively proliferate. After 3 months, the cells were tested for sensitivity to lapatinib and there was no discernible difference in lapatinib or trastuzumab sensitivity between the treatment-naïve and lapatinib-conditioned cells, suggesting that the conditioned cells were equally sensitive to lapatinib as lapatinib-naïve HCC1419 cells (Figure 6B).

## 3. Discussion

To elucidate potential pathways of resistance to HER2 targeted TKIs, we examined the effect of continuous in vitro lapatinib exposure over 1–4 weeks on breast cancer cells. Lapatinib concentrations selected were in the range of the steady state plasma lapatinib concentrations achieved in patients [23]. Distinct morphological changes were observed in lapatinib-treated cells, including increased cell size and development of vacuoles, leading us to hypothesize that the cells were senescent, as these, coupled with SA-β-gal activity, detectable at pH 6.0, are among the hallmarks of senescence [16]. Strong SA-β-gal activity was observed in four HER2 positive lapatinib-sensitive breast cancer cell lines following lapatinib treatment, and not in lapatinib-resistant or HER2 negative cells. In addition to increased SA-β-gal activity, the expression of senescence markers p15 and p27 [24,25], were significantly increased in lapatinib-treated cells. In contrast, lapatinib-induced senescence was not associated with increased expression of p21 or p16, which are typically associated with oncogene-induced senescence [26]. This is not the first report of senescence in the absence of p16, p21 and/or p53 activity; downregulation of p300 histone acetyltransferase activity induces senescence that is independent of p16, p21 and p53 [27] and Aurora kinase inhibition induced senescence in a p16 null cell line [28].

Although the tumor suppressor p53 has been reported to be critical for the establishment of OIS in human cells [18], studies have shown that transformed cells that lack p53 retain the capacity to develop TIS in response to anti-cancer agents [19,29]. Intriguingly, we report lapatinib-induced senescence in cells that are p53 negative, as well as p53 mutant cells, suggesting that lapatinib-induced senescence is not dependent on wt p53 activity. In HCC1419 cells transfected with wt p53, lapatinib treatment killed the majority of the cells with no evidence of increased SA-β-gal activity. This is most likely due to apoptosis induction as previous studies suggest that over a certain threshold of p53 expression, cells are more likely to undergo apoptosis [30]. In p53 mutant (gain of function) SKBR3 cells, treatment with lapatinib in combination with pifthrin, an inhibitor of p53 activity, resulted in an increase in SA-β-gal activity within 1 week, with no SA-β-gal activity in cells treated with either agent alone. In contrast, treatment with APR-246, which restores wt p53 activity [31], resulted in reduced SA-β-gal activity and increased subG1 arrest, suggesting increased apoptosis. These results suggest that low wt or mutant p53 activity contributes to induction of lapatinib-induced senescence, whereas wt p53 may facilitate lapatinib-induced apoptosis. There is also evidence to suggest that induction of TIS is dependent on the concentration of the inducing agent. Lower concentrations may trigger a senescence response without activating the caspase cascade that commits the cell to apoptosis [29]. High dose doxorubicin induced apoptosis in cardiomyocytes, whereas low dose doxorubicin induced TIS [32]. Consistent with this result, HCC1419 cells treated with low concentrations of lapatinib induced stronger SA-β-gal activity compared to concentrations of lapatinib in excess of 500 nM. Taken together these results suggest that lapatinib induced senescence may be dose dependent and modulated by p53 activity.

To determine if other HER2-targeted agents can also induce senescence we tested trastuzumab and the second-generation pan-HER TKIs, neratinib and afatinib [33,34]. Treatment of HCC1419 cells with either neratinib or afatinib induced senescence. However, single agent trastuzumab did not induce senescence in HCC1419 cells, which are innately resistant to trastuzumab or in SKBR3 cells, which are sensitive to trastuzumab. Interestingly, trastuzumab in combination with lapatinib induced senescence in both cell lines, suggesting that trastuzumab treatment does not prevent lapatinib-induced senescence. All three TKIs also target EGFR suggesting that inhibition of EGFR may also play a role in the TKI-induced senescence. Indeed, EGFR inhibition by gefitinib has previously been shown to induce senescence in lung cancer cells [35,36]. However, lapatinib, which also inhibits EGFR, did not induce senescence in MCF7 cells, which express EGFR [37]. In addition, we found that inhibition of either MAPK or PI3K/AKT signaling induces senescence in HCC1419 cells. These results suggest that TKI induced-senescence in HER2 positive breast cancer cells may require inhibition of both HER2 and EGFR kinase activity, to completely block signaling through either the MAPK or AKT pathways.

An important factor that differentiates TIS from replicative senescence is the ability of the cells to escape from senescence and re-enter the cell cycle. An emerging body of evidence suggests that cells can reverse or escape TIS; a small fraction of chemotherapy-induced senescent lung carcinoma cells can bypass senescence and re-enter the cell cycle [38], and induction of senescence by cisplatin has been suggested as a gateway for cells to avoid DNA damage and develop resistance [39]. Lapatinib-induced senescence in HER2 positive cells was reversed upon removal of lapatinib from the culture media. Interestingly, senescence induced by neratinib or afatinib was not reversed following removal of the agents. Both neratinib and afatinib are irreversible TKIs, in contrast to lapatinib, which binds reversibly to the HER kinase domains. Once bound, irreversible inhibitors do not readily dissociate, allowing inhibition to continue even after the inhibitor has been removed from the culture media [40].

Senescence induction may contribute to the results of the NeoALTTO and ALTTO trials. Both trials compared lapatinib and trastuzumab alone and in combination (in conjunction with chemotherapy). In the neoadjuvant trial (NeoALTTO), lapatinib and trastuzumab doubled the pathologic complete response (pCR) rate compared with trastuzumab alone [41]. However, there was no difference in event-free or overall survival (OS) [42]. In the ALTTO trial, patients received adjuvant anti-HER2 therapy for 1 year and the primary endpoint of the study was disease-free survival (DFS) at four years. Addition of lapatinib to trastuzumab failed to show superiority compared to trastuzumab treatment [43]. We found that long-term continuous exposure to lapatinib resulted in senescence, and even after 6 months of exposure, removal of lapatinib from the media resulted in the cells resuming active proliferation and the resulting cell population showed identical sensitivity to lapatinib as treatment naïve cells. Our data suggest that a small subset of tumor cells may become senescent, or senescent cells may be selected for, during lapatinib treatment and then begin active proliferation again when a patient ceases lapatinib therapy. This may explain why the promising pCR for patients treated with trastuzumab and lapatinib therapy in the NeoALTTO trial did not extend into an overall DFS or OS benefit. It also suggests that patients whose HER2 positive breast cancer recurs after completing lapatinib treatment may benefit from re-treatment with lapatinib. Once a patient develops resistance to an agent, either through progression while receiving therapy or a relapse after therapy has been discontinued, the usual clinical strategy is to initiate a different therapy, the assumption being that the previously used agent will be ineffective (reviewed in [44]). However, drug re-challenge regimes have been successfully used to treat patients with progressive and/or recurring disease, including tamoxifen [45] or anthracyclines for breast cancer [46] and platinum-based therapies for ovarian cancer [47].

These exciting in vitro results showing HER2-TKI induced senescence in breast cancer cells will require further testing, including but not limited to (i) examining additional markers of senescence (e.g., trimethyl-K4 histone H3), (ii) examining TKI-treated breast cancer xenograft tissue samples and critically in TKI-treated patient samples to thoroughly evaluate senescence induction as a novel mechanism of action and potential mechanism of resistance to HER2 targeted TKIs.

## 4. Materials and Methods

### 4.1. Cell Lines and Reagents

The EFM-192A (ACC258) cell line was obtained from the German Tissue Repository DSMZ; while the SKBR3 (HTB-30), HCC1419 (CRL-2326), MDA-MB-361 (HTB-27), MDA-MB-453 (HTB-131) and MCF7 (HTB-22) cell lines were obtained from the American Type Culture Collection. All cells were cultured in RPMI-1640 with 10% fetal bovine serum (FBS). Cell lines were routinely tested for mycoplasma and were authenticated by short tandem repeat typing (Source BioScience, Nottingham, United Kingdom). Solutions (10 mM) of lapatinib, neratinib and afatinib (Sequoia Research Products, Pangbourne, United Kingdom) and pifithrin (Sigma, St. Louis, MO, USA) were prepared in dimethyl sulfoxide (Sigma) and APR-246 (Tocris Bioscience, Bristol, United Kingdom) was prepared in sterile dH_2_O. Trastuzumab was obtained from St. Vincent’s University Hospital, Dublin, Ireland.

### 4.2. Growth Inhibition Assays

To test for lapatinib sensitivity, cells were seeded in 24-well plates and incubated for 24 h, then treated with varying concentrations of lapatinib (100, 150, 200, 250, 300, 400 or 500 nM, or 1 μM depending on the cell line) or with 100 nM of trastuzumab for 4 or 5 days (see legends for individual experimental details). Cells were trypsinized and counted using ViaCount (Millipore, Burlington, MA, USA) on the Guava EasyCyte (Millipore) in triplicate. Percentage growth inhibition was calculated relative to untreated controls.

### 4.3. Senescence-Associated β-Galactosidase Activity Assay

Cells were seeded into 25 mm^3^ flasks and after 24 h drugs were added for various time points. Drug containing media was replenished every 3–4 days throughout the treatment process. β-galactosidase (SA-β-gal) activity was compared between treated and untreated cells using a SA-β-gal staining kit (Cell Signaling Technology, Danvers, MA, USA). Representative images were captured using unbiased cell selection at 400× magnification (unless otherwise stated). For the lapatinib dose response assay, the intensity of SA-β-gal staining was estimated using ImageJ software (downloaded from imagej.net). Briefly, the mean gray value for each image was calculated and the average value of triplicate control images was subtracted from the value of each treatment image which was then averaged. A similar analysis was also performed using the color histogram data whereby the mean intensity of the blue channel was compared across samples and reported relative to control images.

### 4.4. Cell Cycle Assay

In a 24 well plate, 1 × 10^4^ cells were seeded per well and incubated at 37 °C. After 24 h, lapatinib (250 nM) and/or APR-246 (5 µM) were added to each well. Following 5 days incubation, media was collected and the wells were rinsed with phosphate buffered saline (PBS). Cells were trypsinized, added to collected media and centrifuged at 300× *g* for 5 min. PBS (150 μL) was added and transferred to a round bottom 96-well plate. After centrifuging at 300× *g* for 5 min, the supernatant was removed, leaving approximately 15 μL per well. The cells were resuspended and 200 μL of ice-cold 70% ethanol was added. After fixing for 2 h at −20 °C and centrifugation at 450× *g* for 5 min, cells were washed with 200 μL of PBS and centrifuged again at 450× *g* for 5 min. PBS was removed and 200 μL of Guava Cell Cycle reagent was added. The resuspended cells were incubated at room temperature in the dark for 30 min, then analyzed on the Guava EasyCyte (Millipore).

### 4.5. RNA Extraction and qRT-PCR Analysis

RNA was extracted using Tri-reagent (Sigma) according to the manufacturer’s instructions. RNA (1 μg) was used for cDNA synthesis using random primers and a cDNA Reverse Transcription Kit (Applied Biosystems, Foster City, CA, USA). Quantitative PCR (qPCR) analysis was carried out on a ABI7900HT fast system (Applied Biosystems) using Taqman Universal PCR Master Mix (Applied Biosystems), according to manufacturer’s instructions for p15 (HS00793225_m1), p16 (HS04189686_m1), p21 (HS00355782_m1) and p27 (HS00153277_m1), with GADPH (HS02758991_g1) as an endogenous control (Applied Biosystems).

### 4.6. Western Blot Analysis

Protein (30 µg) was separated on polyacrylamide gels (Lonza, Basel, Switzerland), transferred to nitrocellulose membranes (Invitrogen, Carlsbad, CA, USA) and blocked for 1 h in blocking solution (2.5% milk powder (Biorad, Hercules, CA, USA) in PBS containing 0.1% Tween-20 (PBS-T)). Membranes were incubated at 4 °C overnight with primary antibody (1:1000) in blocking solution using antibodies against p53 and β-actin (Cell Signaling Technology). Following PBS-T washes, the membrane was incubated for 1 h in anti-rabbit secondary antibody (Sigma). Following washing, detection was performed using Luminol (SantaCruzBiotechnology, Dallas, TX, USA) or ECL Advance (GE Healthcare, Chicago, IL, USA).

### 4.7. p53 Overexpression

A wild-type p53 plasmid (ATCC) was sub-cloned into a pcDNA3.1 vector as previously described [48]. The p53 expressing vector was transfected into HCC1419 cells using lipofectin (Invitrogen), alongside an empty vector control transfection. Transfected cells were selected using 200 µg/mL zeocin for 72 h.

## 5. Conclusions

In summary, we present a novel mechanism of lapatinib action: induction of senescence characterized by increased SA-β-gal activity and increased expression of p15 and p27. Lapatinib-induced senescence is stable in the continuous presence of the inhibitor even up to six months, while removal of the inhibitor releases cells from senescence, allowing them to proliferate with retained sensitivity to lapatinib. Taken together these data provide novel insights into the mechanisms of lapatinib action and the pathways to lapatinib resistance and suggest new therapeutic strategies to improve the response to HER2-targeted therapies.

## Supplementary Materials

The following are available online at http://www.mdpi.com/2072-6694/11/2/197/s1, Figure S1: Quantification of the images provided in Figure 1E, Figure S2: Effect of PI3K/AKT and ERK inhibition on SA-β-gal activity, Figure S3: Continuous treatment of HCC1419 cells with lapatinib results in sustained SA-β-gal activity, Table S1: p16 expression in breast cancer cell lines.
