# Supplementary material for: HER2-Targeted Tyrosine Kinase Inhibitors Cause Therapy-Induced-Senescence in Breast Cancer Cells
Source: Cancers (Basel). 2019 Feb 8;11(2):197. doi: 10.3390/cancers11020197 (PMC6406301; doi:10.3390/cancers11020197)
Supplement: Supplementary file 1 [file cancers-11-00197-s001.pdf]

cancers

MDPI

# Supplementary Material: HER2-Targeted Tyrosine Kinase Inhibitors Cause Therapy-Induced-Senescence in Breast Cancer Cells

**Martina S.J. McDermott, Neil Conlon, Brigid C. Browne, Adam Szabo, Naoise C. Synnott, Neil A. O'Brien, Michael J. Duffy, John Crown and Norma O'Donovan**

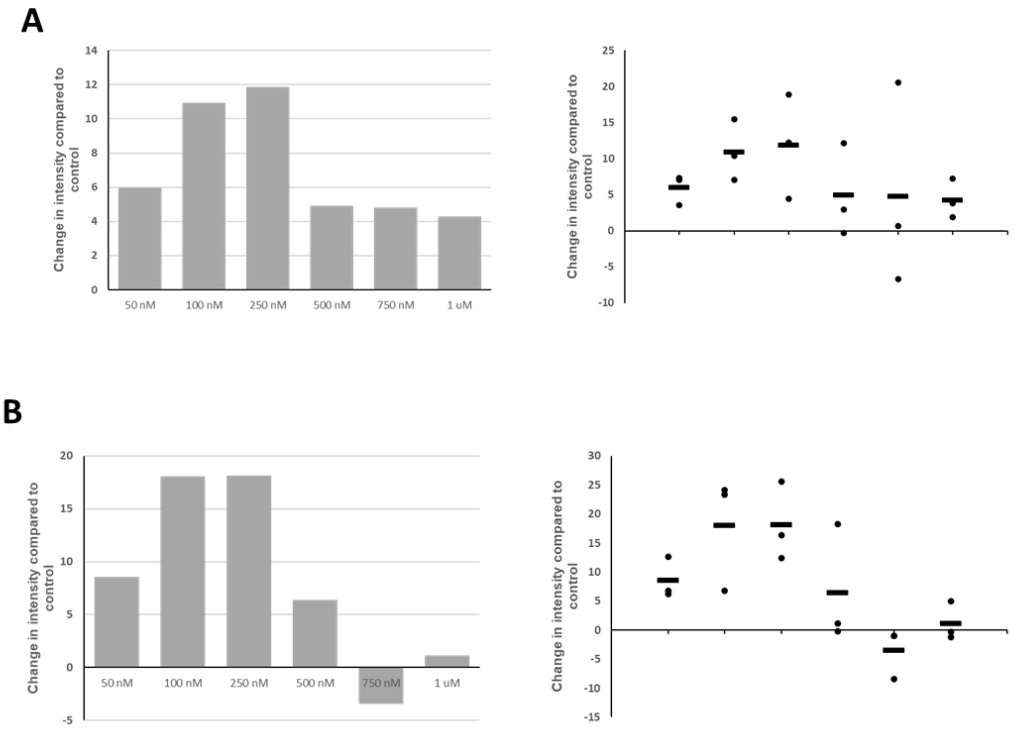


**Figure S1.** Quantification of the images provided in Figure 1E, whereby HCC1419 cells were treated with a range of lapatinib concentrations for 1 week. (**A**) Using ImageJ software the gray mean intensity value for each image was calculated and the average value of triplicate control images was subtracted from the value of each treatment image which was then averaged. (**B**) A similar analysis was also performed using the color histogram analysis whereby the mean intensity of the blue channel was compared across samples and reported relative to control images. In both sets of analyses, the graph on the right shows each individual intensity score represented by a black dot together with the average intensity represented by the black dash; this average intensity value is depicted in the graph on the left.

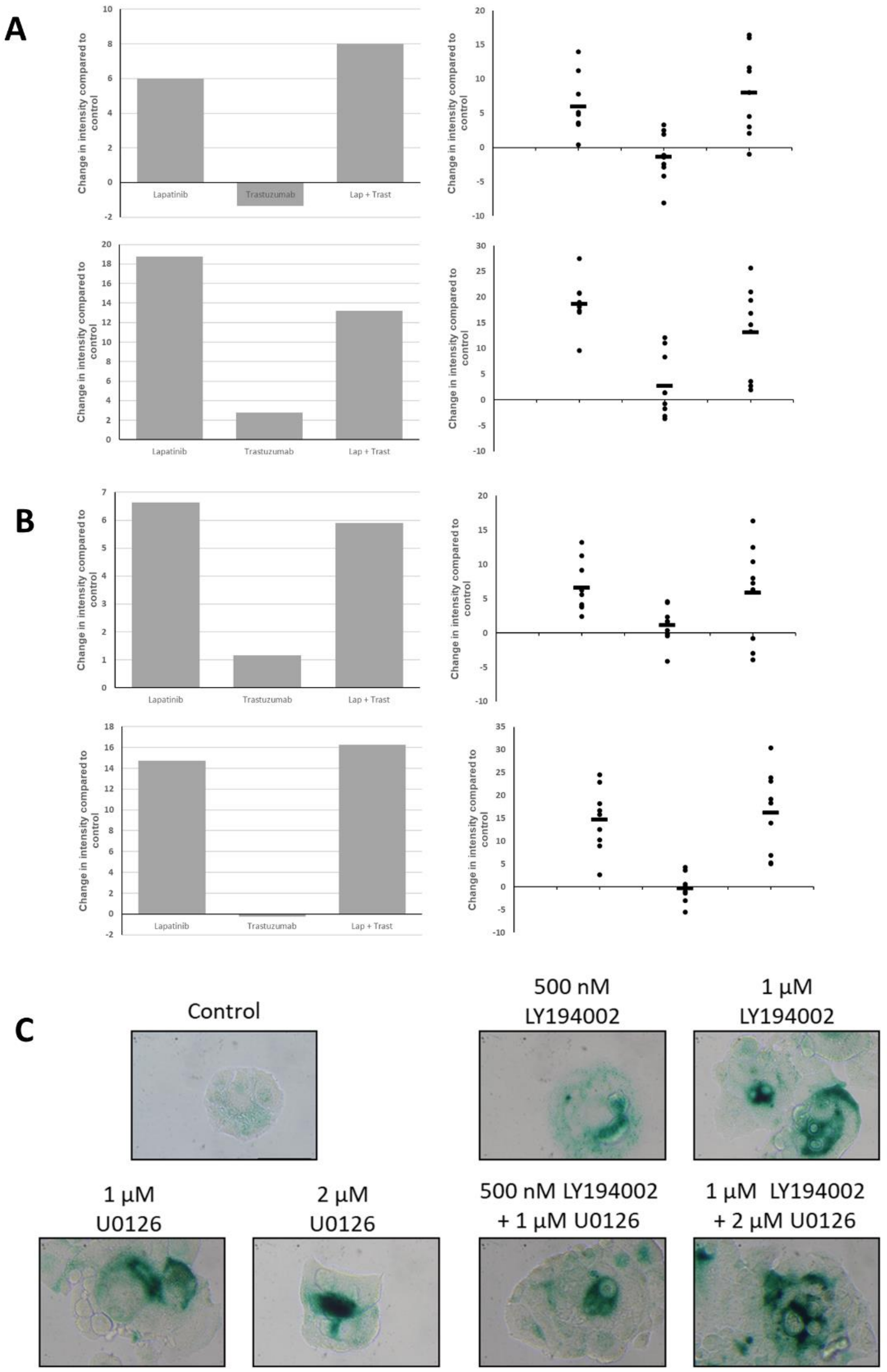


**Figure S2.** Quantification of the images provided in Figure 4B,C. Using ImageJ software the gray (top) and blue (bottom) mean intensity value for each image was calculated and the average value of triplicate control images was subtracted from the value of each treatment image which was then averaged for (**A**) HCC1419 and (**B**) SKBR3 cells. In both sets of analyses, the graph on the right shows each individual intensity score represented by a black dot together with the average intensity represented by the black dash; this average intensity value is depicted in the graph on the left. (**C**) Effect of PI3K/AKT and ERK inhibition on SA-β-gal activity. HCC1419 cells were treated for 1 week with LY294002 (500 nM and 1 µM) or U0126 (1 µM and 2 µM) alone and in combination, Cells were fixed and tested for SA-β-gal activity. Images taken at 400X magnification.

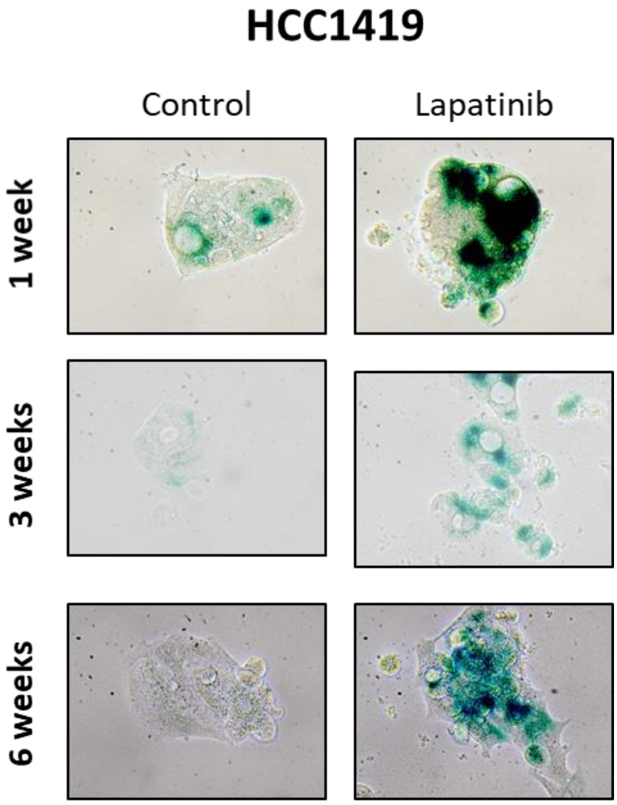


**Figure S3.** Continuous treatment of HCC1419 cells with lapatinib results in sustained SA-β-gal activity. HCC1419 cells were treated with 250 nM lapatinib twice weekly for 6 weeks. Cells were fixed and tested for SA-β-gal activity after 1 week, 3 weeks and 6 weeks of treatment weekly intervals. Images taken at 400X magnification.

**Table 1.** Measurement of p16 expression levels in multiple breast cancer cell lines by RT-PCR.

| Cell Line | p16 Levels |
|---|---|
| HCC1419 | undetectable |
| SKBR3 | undetectable |
| MBA-MB-361 | undetectable |
| EFM-192A | undetectable |
| MDA-MB-453 | undetectable |
| MCF7 | undetectable |
| JIMT-1 | low [1] |
| UACC-732 | low [1] |

[1] Included as a positive control for p16 expression.

© 2019 by the authors. Licensee MDPI, Basel, Switzerland. This article is an open access article distributed under the terms and conditions of the Creative Commons Attribution (CC BY) license (http://creativecommons.org/licenses/by/4.0/).
